# Cystatin C in Alzheimer's disease

**DOI:** 10.3389/fnmol.2012.00079

**Published:** 2012-07-06

**Authors:** Gurjinder Kaur, Efrat Levy

**Affiliations:** Departments of Psychiatry, Biochemistry, and Molecular Pharmacology, Center for Dementia Research, Nathan S. Kline Institute, New York University School of Medicine, OrangeburgNY, USA

**Keywords:** cystatin C, Alzheimer's disease, cerebral amyloidosis, amyloid, Aβ, neurodegeneration

## Abstract

Changes in expression and secretion levels of cystatin C (CysC) in the brain in various neurological disorders and in animal models of neurodegeneration underscore a role for CysC in these conditions. A polymorphism in the CysC gene (*CST3*) is linked to increased risk for Alzheimer's disease (AD). AD pathology is characterized by deposition of oligomeric and fibrillar forms of amyloid β (Aβ) in the neuropil and cerebral vessel walls, neurofibrillary tangles composed mainly of hyperphosphorylated tau, and neurodegeneration. The implication of CysC in AD was initially suggested by its co-localization with Aβ in amyloid-laden vascular walls, and in senile plaque cores of amyloid in the brains of patients with AD, Down's syndrome, hereditary cerebral hemorrhage with amyloidosis, Dutch type (HCHWA-D), and cerebral infarction. CysC also co-localizes with Aβ amyloid deposits in the brains of non-demented aged individuals. Multiple lines of research show that CysC plays protective roles in AD. *In vitro* studies have shown that CysC binds Aβ and inhibits Aβ oligomerization and fibril formation. *In vivo* results from the brains and plasma of Aβ-depositing transgenic mice confirmed the association of CysC with the soluble, non-pathological form of Aβ and the inhibition of Aβ plaques formation. The association of CysC with Aβ was also found in brain and in cerebrospinal fluid (CSF) from AD patients and non-demented control individuals. Moreover, *in vitro* results showed that CysC protects neuronal cells from a variety of insults that may cause cell death, including cell death induced by oligomeric and fibrillar Aβ. These data suggest that the reduced levels of CysC manifested in AD contribute to increased neuronal vulnerability and impaired neuronal ability to prevent neurodegeneration. This review elaborates on the neuroprotective roles of CysC in AD and the clinical relevance of this protein as a therapeutic agent.

## Introduction

CysC also known as γ-trace, is a basic protein (Hochwald et al., 1967), originally identified in human CSF and subsequently, also found in all other mammalian body fluids and tissues (Bobek and Levine, 1992; Turk et al., 2008). CysC is highly abundant in brain tissue (Hakansson et al., 1996), expressed by neurons, astrocytes, and microglial cells in the brains of different species (Yasuhara et al., 1993; Palm et al., 1995; Miyake et al., 1996). CysC plays a variety of biological roles, ranging from anti-viral and anti-bacterial properties (Bobek and Levine, 1992), bone resorption (Lerner and Grubb, 1992), tumor metastasis (Huh et al., 1999; Taupin et al., 2000), modulation of inflammatory responses (Warfel et al., 1987; Bobek and Levine, 1992), cell proliferation and growth (Sun, 1989; Tavera et al., 1992), and astrocytic differentiation during mouse brain development (Kumada et al., 2004). Involvement of CysC has been shown in various diseases ranging from cancer to neurodegenerative disorders. Multiple studies have demonstrated changes in CysC concentrations in serum associated with a variety of conditions, such as chronic kidney disease, urinary infection, cancer, hypertension, cardiovascular disease, rheumatoid arthritis, glucocorticoid treatment, thyroid function, and aging (reviewed in Filler et al., 2005). CysC concentration in specific tissues and body fluids can serve as a marker for a variety of diseases, disease progression, and the effect of therapy.

CysC is also implicated in the processes of neuronal degeneration and repair of the nervous system (reviewed in Gauthier et al., 2011). CysC was originally identified as an inhibitor of cysteine proteases such as cathepsins required for housekeeping function during protein turnover (Turk et al., 2000). Imbalance between active proteases and their endogenous inhibitors may lead to uncontrolled proteolysis, which has been associated with different neurological diseases (Nakamura et al., 1991). The involvement of proteases and their inhibitors in the processes of neuronal degeneration and repair of the nervous system is reviewed in (Tizon and Levy, 2006).

The majority of CysC in the CSF is produced by the choroid plexus (Tu et al., 1992). CysC CSF levels in normal brain were found to be five times higher than plasma levels suggesting a potential physiological role of CysC in the brain (Grubb, 1992). Alterations in CysC levels in the CSF in neurodegenerative diseases have been documented (Pasinetti et al., 2006; Mares et al., 2009; Mori et al., 2009; Tsuji-Akimoto et al., 2009; Yang et al., 2009; Maetzler et al., 2010). For example, CysC has shown a great diagnostic potential as a biomarker for Amyotropic Lateral Sclerosis (ALS), a fatal neuromuscular disease characterized by progressive motor neuron degeneration (Wilson et al., 2010). CysC levels in the CSF of ALS patients were significantly reduced as compared to healthy controls (Pasinetti et al., 2006; Tsuji-Akimoto et al., 2009; Wilson et al., 2010). In addition, the direction of the longitudinal change in CSF CysC levels correlated with the rate of ALS disease progression, and initial CSF CysC levels were predictive of patient survival, suggesting that CysC may function as a surrogate marker of disease progression and survival (Wilson et al., 2010). CysC is also linked to ALS histopathologically, as it is one of only two known proteins that localize to Bunina bodies, small intraneuronal inclusions contained in degenerating motor neurons, which are a specific neuropathologic feature of ALS (Okamoto et al., 2008). Similarly, it was shown that CysC levels in the CSF of AD patients are lower compared to non-demented individuals (Simonsen et al., 2007; Hansson et al., 2009). Furthermore, specific neuronal cell populations in the brains of patients with AD showed an enhanced CysC expression (Deng et al., 2001; Levy et al., 2001). Changes in the levels of CysC in the CSF and brain in various neurodegenerative diseases suggest important roles for the secreted protein in these disorders. In support of such a role, CysC level dysregulation was also observed in animal models of neurodegenerative conditions caused by facial nerve axotomy (Miyake et al., 1996), noxious input to the sensory spinal cord (Yang et al., 2001), perforant path transections (Ying et al., 2002), hypophysectomy (Katakai et al., 1997), transient forebrain ischemia (Palm et al., 1995; Ishimaru et al., 1996), photothrombotic stroke (Pirttila and Pitkanen, 2006), and induction of epilepsy (Aronica et al., 2001; Hendriksen et al., 2001; Lukasiuk et al., 2002). Augmented CysC expression in the neurodegenerative states puts forward two conflicting theories whether enhanced CysC expression is causing or further exacerbating already initiated neurodegenerative changes or, alternatively, it is an endogenous neuroprotective response to the disease (reviewed in Gauthier et al., 2011). This review focuses on the roles of CysC in the pathological processes of AD.

## CysC and Alzheimer's disease

AD pathology is characterized by formation of amyloid deposits in the brain composed mainly of Aβ, a processing product of the amyloid β precursor protein (APP), aggregation of neurofibrillary tangles composed mainly of hyperphosphorylated tau, loss of neurons with accelerated atrophy of specific brain areas and decreased synapse number in surviving neurons. While it was demonstrated that fibrillar Aβ plays a central role in neurotoxicity in AD brains (for review see Butterfield and Boyd-Kimball, 2004), both *in vitro* and *in vivo* reports describe a potent neurotoxic activity for soluble, nonfibrillar, oligomeric assemblies of Aβ (for reviews see Klein et al., 2001; Walsh and Selkoe, 2004). In this section, we discuss the involvement of CysC in AD as suggested by immunohistochemical, genetic, and biochemical studies.

### CysC co-deposition with amyloid β

The involvement of cystatins in AD was originally suggested due to their co-localization with amyloid plaques. CysC was the first cystatin found co-localized with Aβ in amyloid-laden vascular walls, and in senile plaque cores of amyloid in brains of patients with AD, Down's syndrome, HCHWA-D, intracranial hemorrhage, cerebral infarction, and of elderly subjects without any neurological disorder (Maruyama et al., 1990; Vinters et al., 1990; Itoh et al., 1993; Haan et al., 1994; Levy et al., 2001). Abundant cystatin A (CysA) and cystatin B (CysB), also called stefin B, were demonstrated in senile plaques in the brain of AD patients (Ii et al., 1993; Bernstein et al., 1994).

The deposition of fibrillar protein aggregates in the walls of arteries, arterioles, and sometimes capillaries and veins of the central nervous system is known as cerebral amyloid angiopathy (CAA) (Nagai et al., 2008). Hereditary cerebral hemorrhage with amyloidosis, Icelandic type (HCHWA-I) (Arnason, 1935; Gudmundsson et al., 1972), also called hereditary cystatin C amyloid angiopathy (HCCAA; Olafsson et al., 1996), is an autosomal dominant form of CAA. Amyloid deposition in cerebral and spinal arteries and arterioles of HCHWA-I patients leads to recurrent hemorrhagic strokes causing serious brain damage and eventually fatal stroke (Gudmundsson et al., 1972). The amyloid deposited is composed mainly of a Leu68Gln variant of CysC (Cohen et al., 1983; Ghiso et al., 1986; Palsdottir et al., 1988; Levy et al., 1989; Abrahamson et al., 1990). A heterozygous point mutation, identical to that found in the *CST3* gene of these patients, was also identified in a Croatian man with CAA and intracerebral hemorrhage (Graffagnino et al., 1995). Thus, sporadic CAA in some patients may be associated with mutations in the *CST3* gene (Graffagnino et al., 1995; McCarron et al., 2000).

Amyloid β usually accumulates both in cerebral blood vessels and in brain parenchyma as amyloid plaques. However, in some cases Aβ deposits predominantly in the cerebral vasculature (Vinters, 2001). The factors leading to vascular rather than parenchymal amyloid deposition are unknown and it is unclear when CAA leads to hemorrhage. A role for CysC in CAA-related hemorrhage is implicated from immunohistochemical studies that revealed co-localization of CysC and Aβ in amyloid-laden vascular walls (Maruyama et al., 1990; Vinters et al., 1990; Itoh et al., 1993; Haan et al., 1994). It was reported that only patients showing co-localization of CysC and Aβ immunoreactivity in their diseased cerebral vessels suffered fatal subcortical hemorrhages (Maruyama et al., 1990). The degree of cerebrovascular amyloid deposition in these patients was also greater than in patients without cerebral hemorrhages. Studies were conducted to find out whether CysC exists as amyloid fibrils or as unpolymerized CysC absorbed onto or trapped within the bundles of Aβ amyloid fibrils. ELISA analysis of crude amyloid fibrils isolated from cerebral blood vessels of one patient revealed that CysC and Aβ have been included at the ratio of about 1:100 (Nagai et al., 1998). In another case of sporadic CAA, isolation and chemical analysis of amyloid fibril proteins from leptomeningeal vessels revealed that while Aβ was fibrillar, CysC was soluble (Maruyama et al., 1992). It has been suggested that CysC deposition occurs secondarily to Aβ deposition and may increase the predisposition to cerebral hemorrhages (Itoh et al., 1993).

CysC also co-localizes with Aβ deposits in the brains of animal models of cerebral amyloidosis. Co-localization of Aβ and CysC was demonstrated in vascular and parenchymal deposits in the brains of aged rhesus monkeys and in vascular amyloid in brains of aged squirrel monkeys (Wei et al., 1996). Non-human primates are good models to study cerebral changes that occur through aging. Neuropathologies characteristic of AD and normal aging in humans were also found in senescent non-human primates (Wisniewski and Terry, 1973; Walker et al., 1990; Price et al., 1994). In aged rhesus monkeys (*Macaca mulatta*), amyloid deposition predominates in senile plaques with relatively minor vascular involvement. However, cerebrovascular deposits in aged squirrel monkeys (*Saimiri sciureus*), usually are more conspicuous than senile plaques (Walker et al., 1990). Sequence analysis of rhesus and squirrel monkey CysC cDNA revealed that squirrel monkey has Met at position 68, which is Leu in the rhesus and wild-type human CysC and Gln in HCHWA-I patients (Wei et al., 1996). An additional difference between squirrel and rhesus monkeys in CysC sequence was found at position 10, a residue that was shown to affect the specificity of the inhibitor for different cysteine proteases (Lindahl et al., 1994). The species-specific CysC sequences in humans, rhesus, and squirrel monkeys may be responsible for the variability of the amyloid deposits observed.

In order to elucidate the role of increased expression of this protein *in vivo*, CysC transgenic mice were generated (Pawlik et al., 2004). These mice express either human wild-type or the Leu68Gln variant CysC genes under the transcriptional control of its own promoter (Levy et al., 1989), overexpressing the transgene along with its endogenous counterpart in the appropriate tissues (Pawlik et al., 2004). Lines of mice expressing various levels of the transgene in the brain were selected. All selected lines had very high concentrations of the transgene in the blood. None of the mice had amyloid deposits either in the vessel walls or in the neuropil. Neuropathological examination of dead or ailing aged transgenic mice revealed some mice with cerebral or subarachnoid hemorrhages (Pawlik et al., 2002). Conversely, no hemorrhages were observed in their non-transgenic siblings. These data demonstrate that elevated brain and/or blood levels of CysC can cause hemorrhagic strokes in the absence of vascular amyloid deposits. It has been shown that the risk of cerebral hemorrhage in the brains of aged individuals and AD patients increases when high levels of CysC are present in cerebrovascular Aβ deposits. These findings suggest that binding of CysC to Aβ in the vasculature, resulting in local accumulation of the protease inhibitor, may contribute to hemorrhages.

CysC also co-localizes with Aβ deposits in the parenchyma and vasculature in brains of transgenic mice overexpressing human APP (Levy et al., 2001; Steinhoff et al., 2001). A striking increase in CAA with aging was found in the APP transgenic mouse line APP23 (Winkler et al., 2001). Antibodies to CysC revealed appreciable staining of cerebrovascular amyloid in these mice. Similar to CysC staining of Aβ deposits in human brains, the CysC immunoreactivity was restricted to a subpopulation of amyloid-laden vessels and was clearly less intense than Aβ staining (Levy et al., 2001; Steinhoff et al., 2001; Winkler et al., 2001).

Wild-type CysC co-localization with amyloid, other than Aβ, was observed in a variety of disorders, such as hereditary gelsolin amyloidosis (familial amyloidosis, Finnish type; Kiuru et al., 1999; Kiuru-Enari et al., 2002) and familial CAA, British type (Ghiso et al., 1995). Thus, CysC may play a role in CAA and hemorrhage in a variety of diseases that involve deposition of heterogeneous types of amyloid proteins.

### Intracellular co-localization of CysC with Aβ in the brain

Immunohistochemical analyses have shown intense CysC immunoreactive neurons and activated glia in the cerebral cortex of some aged human cases and of all AD patients (Yasuhara et al., 1993; Deng et al., 2001). High neuronal staining of CysC in AD brains was primarily limited to pyramidal neurons in cortical layers III and V (Deng et al., 2001; Levy et al., 2001). The regional distribution of CysC neuronal immunostaining duplicated the pattern of neuronal susceptibility in AD brains: the strongest staining was found in the entorhinal cortex, in the hippocampus, and in the temporal cortex; fewer pyramidal neurons were stained in the frontal, parietal, and occipital lobes (Deng et al., 2001). Using an end-specific antibody to the carboxyl-terminus of Aβ_42_, intracellular immunoreactivity was observed in the same neuronal subpopulation (Levy et al., 2001). These data showed that Aβ_42_ accumulates in a specific population of pyramidal neurons in the brain, the same cell type in which CysC is highly expressed.

### Low concentration of CysC in CSF and plasma of AD patients

Low levels of serum CysC precede clinically manifested AD in elderly men free of dementia at baseline and may be a marker of future risk of AD (Sundelof et al., 2008). Clinically diagnosed AD patients also showed a reduction in the CSF levels of CysC compared to controls (Hansson et al., 2009). Interestingly, CysC levels were positively correlated with both tau and Aβ_42_ levels in the CSF, independent of age, gender, and apolipoprotein E (APOE) genotype (Sundelof et al., 2010). Therefore, it was suggested to measure the change in CysC CSF levels as a biomarker for AD diagnosis (Simonsen et al., 2007; Mares et al., 2009; Zellner et al., 2009; Ndjole et al., 2010; Sundelof et al., 2010; Craig-Schapiro et al., 2011; Perrin et al., 2011). Moreover, analysis of CysC levels in plasma revealed a significant tendency of conversion from mild cognitive impairment to dementia in subjects with CysC levels below the median (CysC lower than 1067 ng/ml; Ghidoni et al., 2010). A large proportion of demented Lewy body disease patients have AD-like pathology, in particular Aβ plaques. Demented Lewy body disease patients also showed decreased CSF CysC levels (Maetzler et al., 2010).

### Linkage of CysC gene polymorphism with AD

Studies were conducted to determine whether the association of CysC with AD could be identified at the genetic level. The *CST3* gene, localized on chromosome 20 (Abrahamson et al., 1989; Saitoh et al., 1989), has three genetically linked base substitutions in the 3′ region (Balbin and Abrahamson, 1991; Balbin et al., 1993). A G73A transition in exon 1 results in Ala/Thr variation in the coding region of *CST3*, within the signal peptide. While the allele containing Ala at that position was called the A allele, the one containing Thr in the same position was called B allele. Several studies have linked *CST3* gene polymorphisms with an increased risk of developing AD (Crawford et al., 2000; Finckh et al., 2000; Beyer et al., 2001; Olson et al., 2002; Lin et al., 2003; Goddard et al., 2004; Cathcart et al., 2005; Bertram et al., 2007) and a possible interaction with APOE genotype was noted. However, some studies have failed to show an association between *CST3* and AD in a German cohort (Dodel et al., 2002), a Dutch sample with early onset AD (Roks et al., 2001), Japanese AD patients (Maruyama et al., 2001), a Finnish population (Helisalmi et al., 2009), and in early onset AD families (Parfitt et al., 1993). Other studies found a connection between the *CST3* polymorphism and AD in Caucasian populations, but not in Asian populations (Hua et al., 2012), including Chinese (Wang et al., 2008). Over-all meta-analyses using this polymorphism have reported *CST3* as a susceptibility gene for AD. For update on the linkage of the *CST3* polymorphism with AD, see the Alzgene Internet site of the Alzheimer Research Forum (http://www.alzforum.org/res/com/gen/alzgene/geneoverview.asp?geneid=66).

Some groups have shown that the A allele of *CST3* may be critical for the development of AD while others have held B allele responsible for that. A multicentric AD population was genetically studied by age at onset and it was found that A allele of *CST3* has an age related increased influence on onset of AD (Crawford et al., 2000). A significant interaction between the homozygous A genotype of *CST3* and age of onset of AD was found, such that in the over 80 years age group this genotype was responsible for a twofold increased risk for the disease. This interaction was independent of the APOE genotype (Crawford et al., 2000). Another study of large European and American populations, with mean age at onset of 73.1 and 75.0 for AD and controls, respectively, showed linkage between the B allele and late onset AD with no synergistic association with APOE allele (Finckh et al., 2000). A synergistic association between the *CST3* and APOE ε4 alleles was found in a Spanish sample. The *CST3* B allele caused a threefold elevated risk of AD before age 70 and there was an eightfold increase in risk for APOE ε4 carriers with this allele (Beyer et al., 2001). In another genetic study the combination of one or two *CST3* B alleles and APOE ε4 carried a 14-fold increased risk for men and 16-fold for women. These risks apply to a shift in risk from ages 65 and older to younger ages (Cathcart et al., 2005). The attempt to determine the association between *CST3* polymorphism and AD or vascular dementia resulted in the associations between *CST3* B genotype and AD patients older than 75, or vascular dementia patients younger than 75 years. A synergistic association of *CST3* and APOE ε4 alleles was observed in predicting vascular dementia patients (Lin et al., 2003).

The amino acid exchange from Ala to Thr at the –2 position for signal peptide cleavage alters the hydrophobicity profile of the signal sequence (Finckh et al., 2000), resulting in a less efficient cleavage of the signal peptide and thus a reduced secretion of CysC (Benussi et al., 2003). The *CST3* gene G73A polymorphism functionally affects CysC plasma levels (Noto et al., 2005) and CysC CSF levels (Maetzler et al., 2010; Yamamoto-Watanabe et al., 2010). Maetzler and colleagues (Maetzler et al., 2010) showed that the BB genotype of the *CST3* gene is associated with reduced CSF CysC levels in patients with Lewy body disease with dementia. Furthermore, a study of the targeting of the Thr haplotype in cultured retinal pigment epithelial and HeLa cells have shown that a proportion of the Thr protein undergoes incorrect trafficking. In contrast to the Ala haplotype that is targeted to the Golgi apparatus, the Thr variant was associated primarily with mitochondria, resulting in a substantial reduction in the efficiency of targeting CysC for secretion (Paraoan et al., 2004). A multicentric electroencephalographic (EEG) study analyzed the effects of *CST3* haplotypes on resting cortical rhythmicity in subjects with AD and mild cognitive impairment. A relationship between the *CST3* Thr haplotype and global neurophysiological phenotype (i.e., cortical delta and alpha rhythmicity) was found. While APOE ε4 affects EEG rhythms in AD (Lehtovirta et al., 1996, 2000; Jelic et al., 1997), the effects of *CST3* polymorphism were independent of APOE ε4 co-presence (Babiloni et al., 2006). A decreased CysC secretion associated with a polymorphism found in the CysC gene, puts forward a mechanism for the increased-risk of late-onset sporadic AD conferred by this polymorphism and suggests that reduced CysC brain concentration may be associated with the disease.

### Altered CysC trafficking and familial AD

Mutations in the presenilin 1 and presenilin 2 genes account for the majority of familial AD (FAD) cases. Two of the mutations in the presenilin 2 gene that are linked to FAD (PS2 M239I and T122R) alter CysC trafficking in mouse primary neurons and cause reduced CysC secretion (Ghidoni et al., 2007). The primary structure of CysC is indicative of a secreted protein and accordingly, it was demonstrated that most of the CysC is targeted extracellularly via the secretory pathway (Wei et al., 1998; Paraoan et al., 2001). Moreover, it was recently shown that CysC is also secreted in association with exosomes (Ghidoni et al., 2011). Full-length APP and APP processing products, including Aβ are also released in association with exosomes. Over-expression of presenilin 2 with the FAD-associated mutations (PS2 M239I and PS2 T122R) resulted in decreased levels of CysC within exosomes (Ghidoni et al., 2011). Assuming a protective role for CysC, as described below, the reduction in CysC levels may represent the molecular factor responsible for the increased risk of AD in FAD patients, the carriers of these mutations.

### Neuroprotection by CysC in AD

Intense CysC immunoreactivity was observed in specific neuronal populations in the cerebral cortex of some aged human cases and of all AD patients (Yasuhara et al., 1993; Deng et al., 2001; Levy et al., 2001). Enhanced CysC expression in specific cell populations is not limited to AD but was also observed in other neurodegenerative conditions, such as epilepsy, ischemia, and progressive myoclonus epilepsy (Palm et al., 1995; Ishimaru et al., 1996; Aronica et al., 2001; Hendriksen et al., 2001; Lukasiuk et al., 2002; Kaur et al., 2010). Contradictory conclusions were reached from multiple studies, suggesting that increased CysC cellular expression in the brain is either associated with the neurodegenerative process, or alternatively is part of a neuroprotective response aimed at prevention of neurodegeneration. In this section of the review we provide a description of different neuroprotective mechanisms activated by CysC. We hypothesize that reduced secretion of CysC into extracellular body fluids can hamper the ability of the brain to prevent neurodegeneration in various pathological conditions.

#### Protection by inhibition of cysteine proteases

*In vitro* experiments have shown that CysC inhibits cathepsins B, H, K, L, and S (for review see Bernstein et al., 1996) and is inactivated by proteolytic degradation by cathepsin D (Abrahamson et al., 1991; Lenarcic et al., 1991). Neuropathological observations suggest an association between CysC and cathepsins B and D in AD (Deng et al., 2001). Pyramidal neurons in layers III and V in the cortex of AD patients have displayed a quantitative increase in cathepsin D immunoreactivity (Cataldo et al., 1995), the same neuronal population that show increased CysC expression (Deng et al., 2001; Levy et al., 2001). Lysosomal cathepsins are involved in neuronal cell death (Cataldo and Nixon, 1990). Intense cytoplasmic labeling of cathepsin B was detected when neurons had become morphologically altered with obvious shrinkage of the cytoplasm (Hill et al., 1997). Enhanced expression of several cathepsins has been documented in response to injuries, similar to those inducing CysC expression upregulation, such as in transient ischemia (Nitatori et al., 1995; Yamashima et al., 1998), and inhibitors of cathepsins B and L reduce neuronal damage in the hippocampus after ischemia (Tsuchiya et al., 1999). An imbalance in the expression of cathepsins and their inhibitors may cause or exacerbate existing neuropathological changes and increased localizes CysC expression may represent an attempt to curb the cathepsin activity.

The *in vivo* role of CysC as an inhibitor was observed by deletion of CysC in knockout mice, resulting in an increased cathepsin B activity (Sun et al., 2008). Another *in vivo* study has demonstrated that CysC can mediate neuroprotection by inhibition of cysteine proteases. A mouse model of an inherited neurodegenerative disorder, the progressive myoclonic epilepsy, has been generated by knocking-out the CysB gene (Pennacchio et al., 1998). CysB deficiency in these mice results in increased cathepsins activity (Kaur et al., 2010). CysC overexpression in CysB knockout mice decreased cathepsin B and D activities in the brain (Kaur et al., 2010). It was demonstrated that clinical symptoms and neuropathologies, including deficient motor coordination, cerebellar atrophy, neuronal loss in the cerebellum and cerebral cortex, and gliosis caused by CysB deficiency, are rescued by CysC overexpression (Kaur et al., 2010). These data show that CysC partially prevents neurodegeneration in CysB knockout mice through inhibition of cathepsins activity.

#### Protection by induction of autophagy

An *in vitro* study of the effect of CysC on cells of neuronal origin under neurotoxic stimuli has shown that CysC protects neuronal cells from death by a mechanism that does not require cathepsin inhibition (Tizon et al., 2010b). Exogenously applied human CysC protected neuronal cells from death in a concentration dependent manner. Moreover, primary cortical neurons isolated from the brains of CysC overexpressing transgenic mice (Pawlik et al., 2004) were protected from spontaneous death induced by culturing and from B27-supplement-deprivation, and cells isolated from CysC knockout mice (Huh et al., 1999) were more sensitive to *in vitro* toxicity compared to cells isolated from brains of wild-type mice (Tizon et al., 2010b). Using multiple methods, it was demonstrated that CysC induces autophagy in cells under basal conditions, and enhances the autophagic activation in cells exposed to nutritional deprivation and oxidative stress (Tizon et al., 2010b). The autophagic pathway consists of sequestration and turnover of organelles and cytoplasm in autophagic vacuoles that following maturation fuse with lysosomes, leading to degradation of their content. CysC induces a fully functional autophagy via the mTOR pathway that includes competent proteolytic clearance of autophagy substrates by lysosomes (Tizon et al., 2010b). Enhanced lysosomal turnover can protect against neurodegeneration and CysC can serve to modulate the efficiency of the autophagic pathway. It remains to be demonstrated that CysC induces autophagy *in vivo* as a protective mechanism in brain injury and in neurodegenerative disorders, such as AD.

#### Protection by inhibition of Aβ oligomerization and amyloid fibril formation

The co-localization of CysC with Aβ in parenchymal and vascular amyloid deposits reflects the involvement of CysC in amyloidogenesis, therefore, the association of CysC with Aβ was determined by Western blot analysis of immunoprecipitated cell lysate or medium proteins, revealing binding of CysC to full-length APP and to secreted soluble APP (Sastre et al., 2004). Deletion mutants of APP localized the CysC binding site to the Aβ region within APP. CysC association with APP resulted in increased secretion of soluble APP but did not affect the levels of secreted Aβ both *in vitro* (Sastre et al., 2004) and *in vivo* in transgenic mice expressing the human CysC gene (Pawlik et al., 2004). The association of CysC with APP was confirmed using a method for the *in vivo* mapping of protein interactions in intact mouse tissue (Bai et al., 2008). CysC does not bind only to Aβ sequences within APP, but also to the peptide itself (Sastre et al., 2004). Analysis of the association of CysC and Aβ demonstrated a specific, saturable and high affinity binding between CysC and both Aβ_1−42_ and Aβ_1−40_ (Sastre et al., 2004). Most importantly, CysC association with Aβ resulted in a concentration dependent inhibition of Aβ amyloid fibril formation (Sastre et al., 2004). *In vitro* studies also demonstrated that CysC association with Aβ inhibits Aβ oligomerization (Selenica et al., 2007; Tizon et al., 2010a). A structural model of the human CysC/Aβ complex using a combination of selective proteolytic excision and high-resolution mass spectrometry identified a specific C-terminal epitope (residues 101–117) as the Aβ-binding region within CysC (Juszczyk et al., 2009).

The anti-amyloidogenic role of CysC was demonstrated *in vivo* in Aβ depositing APP transgenic mice overexpressing human CysC. Several lines of transgenic mice, expressing human CysC either under control sequences of the human CysC gene (Mi et al., 2007), or specifically in cerebral neurons (Kaeser et al., 2007), were crossbred with mice overexpressing human APP. CysC bound to the soluble, non-pathological form of Aβ in the brains and plasma of these mice and inhibited the aggregation and deposition of Aβ plaques in the brain (Kaeser et al., 2007; Mi et al., 2007). However, deletion of CysC in knockout mice resulted in enhanced Aβ degradation (Sun et al., 2008). Unlike a complete deletion of CysC, reduced or enhanced levels of CysC expression affect the aggregation of Aβ, not Aβ levels (Kaeser et al., 2007; Mi et al., 2007).

Investigation of the binding between Aβ and CysC in human central nervous system was conducted by co-immunoprecipitation of CysC and Aβ in brain and CSF from AD patients and controls (Mi et al., 2009). Sequential centrifugation of brain homogenates was used to identify that the cellular fraction contains Aβ/CysC complexes. While CysC binding to soluble Aβ was observed in AD patients and controls, an SDS-resistant, stable CysC/Aβ complex was detected exclusively in brains of neuropathologically normal controls, but not in AD cases. The association of CysC with Aβ in brain from control individuals and in CSF revealed an interaction of these two polypeptides in their soluble form (Mi et al., 2009). The association between Aβ and CysC was shown to prevent Aβ accumulation and fibrillogenesis in experimental systems, arguing that CysC plays a protective role in the pathogenesis of AD in humans and explains why a decrease in CysC concentration caused by the *CST3* polymorphism or by specific presenilin 2 mutations, can lead to the development of the disease. In addition to its anti-amyloidogenic property, CysC directly protects neuronal cells from Aβ toxicity. The extracellular addition of human CysC together with preformed either oligomeric or fibrillar Aβ to cultured primary hippocampal neurons and to a neuronal cell line increased cell survival (Tizon et al., 2010a). The data obtained show that subtle modifications in CysC expression levels in the central nervous system, or possibly in the periphery, affect amyloid deposition and protect from the toxicity of aggregated Aβ.

Aβ interacts not only with CysC (Sastre et al., 2004), but also with CysB (Skerget et al., 2010; Zerovnik et al., 2010). It was shown that CysB binding to Aβ is oligomer specific and that the dimers and tetramers of CysB inhibit Aβ fibril formation (Skerget et al., 2010). Aβ interaction with amyloid proteins is not restricted to CysC, but include transthyretin (Schwarzman et al., 1994, 2004; Choi et al., 2007; Buxbaum et al., 2008), gelsolin (Chauhan et al., 1999), α_2_-macroglobulin (Kuo et al., 2000), and crystallin-αB (Wilhelmus et al., 2006). The interaction between the amyloidogenic proteins and Aβ inhibits Aβ fibril formation (Matsuoka et al., 2003; Sastre et al., 2004; Wilhelmus et al., 2006; Kaeser et al., 2007; Mi et al., 2007; Skerget et al., 2010). Wilhelmus et al. (2007) suggested that “amateur” chaperones that co-localize with the pathological lesions of AD, such as apolipoproteins and heparan sulfate proteoglycans, bind amyloidogenic proteins and may be involved in conformational changes of Aβ and in the clearance of Aβ from the brain via phagocytosis or active transport across the blood-brain barrier. Similarly, interaction between amyloidogenic proteins results in inhibition of amyloid formation and, therefore, has a neuroprotective function in diseases such as AD.

#### Protection by neurogenesis

CysC can also regulate cell proliferation (Sun, 1989; Tavera et al., 1992). In rats undergoing acute hippocampal injury or status epilepticus-induced epileptogenesis, the expression of CysC mRNA and protein are increased in the hippocampus and in the dentate gyrus (Aronica et al., 2001; Hendriksen et al., 2001; Lukasiuk et al., 2002). The time of increased CysC expression was shown to be matching the time of prominent neurogenesis (Parent et al., 1997; Nairismagi et al., 2004). Moreover, the basal level of neurogenesis in the subgranular layer of dentate gyrus was decreased (Taupin et al., 2000; Pirttila et al., 2005) and the proliferation and migration of newborn granule cells in the dentate gyrus were impaired in CysC knockout mice (Pirttila et al., 2005), supporting a role for CysC in neurogenesis. CysC was shown to regulate glial development, as addition of human CysC into the culture medium of primary brain cells increased the number of glial fibrillary acidic protein (GFAP)-positive and nestin-positive cells, as well as the number of neurospheres formed from embryonic brain (Hasegawa et al., 2007). Thus, another mechanism of neuroprotection by CysC might involve induction of neurogenesis.

## Conclusions

Immunohistochemical, genetic, and biochemical studies suggest the involvement of CysC in AD. Immunohistochemical studies have shown that CysC co-localizes with Aβ in amyloid-laden vascular walls, and in senile plaque cores of amyloid and that CysC and Aβ immunoreactivity co-localizes in a specific population of pyramidal neurons that is vulnerable to neurodegeneration in AD. Biochemical studies have shown binding of CysC to Aβ and that this binding prevents Aβ oligomerization, fibril formation, and amyloid deposition. Genetic studies have shown linkage of a CysC gene polymorphism with AD, associated with decreased secretion of CysC. Two FAD-linked presenilin 2 gene mutations alter CysC trafficking and cause reduced CysC secretion. Moreover, low concentrations of CysC were measured in CSF and plasma of AD patients. While some studies have shown that CysC can be toxic to cells under certain conditions, there are reports showing that under other specific conditions, CysC can be protective. While in high concentrations CysC may be toxic to cells, low concentrations may not be sufficient to protect the cells. We hypothesize that protection imparted by CysC is efficient under a limited range of concentrations. Slightly increased CysC expression activates multiple mechanisms of protection. These mechanisms include inhibition of cysteine proteases, induction of autophagy, induction of cell division, and prevention of amyloidogenesis. We hypothesize that in AD, a variety of these mechanisms are activated. However, the reduction in CysC levels may represent the molecular factor responsible for increased risk of AD. These finding propose that a therapy involving enhancing CysC concentration may be developed to prevent, postpone, or halt the disease.

### Conflict of interest statement

The authors declare that the research was conducted in the absence of any commercial or financial relationships that could be construed as a potential conflict of interest.
